# Evolution of self-organized division of labor in a response threshold model

**DOI:** 10.1007/s00265-012-1343-2

**Published:** 2012-03-22

**Authors:** Ana Duarte, Ido Pen, Laurent Keller, Franz J. Weissing

**Affiliations:** 1 Theoretical Biology Group, Centre for Ecological and Evolutionary Studies, University of Groningen, P.O. Box 11103, Groningen, 9700 CC Netherlands; 2 Department of Ecology and Evolution, University of Lausanne, CH-1015 Lausanne, Switzerland; 3 Department of Zoology, University of Cambridge, Downing Street, CB2 3EJ Cambridge, UK

**Keywords:** Response threshold model, Specialization, Emergent properties, Genetic task determination

## Abstract

Division of labor in social insects is determinant to their ecological success. Recent models emphasize that division of labor is an emergent property of the interactions among nestmates obeying to simple behavioral rules. However, the role of evolution in shaping these rules has been largely neglected. Here, we investigate a model that integrates the perspectives of self-organization and evolution. Our point of departure is the response threshold model, where we allow thresholds to evolve. We ask whether the thresholds will evolve to a state where division of labor emerges in a form that fits the needs of the colony. We find that division of labor can indeed evolve through the evolutionary branching of thresholds, leading to workers that differ in their tendency to take on a given task. However, the conditions under which division of labor evolves depend on the strength of selection on the two fitness components considered: amount of work performed and on worker distribution over tasks. When selection is strongest on the amount of work performed, division of labor evolves if switching tasks is costly. When selection is strongest on worker distribution, division of labor is less likely to evolve. Furthermore, we show that a biased distribution (like 3:1) of workers over tasks is not easily achievable by a threshold mechanism, even under strong selection. Contrary to expectation, multiple matings of colony foundresses impede the evolution of specialization. Overall, our model sheds light on the importance of considering the interaction between specific mechanisms and ecological requirements to better understand the evolutionary scenarios that lead to division of labor in complex systems.

**Electronic supplementary material:**

The online version of this article (doi:10.1007/s00265-012-1343-2) contains supplementary material, which is available to authorized users.

## Introduction

Division of labor can be understood as the partitioning of work among specialists in a system, leading to an overall higher performance of the system. The study of division of labor is relevant across several disciplines, such as economics, robotics, and biology, having a central place in the understanding of social evolution.

In eusocial insects, such as ants, bees, wasps, and termites, division of labor among workers in the non-reproductive caste is often considered to be determinant of their ecological success (Hölldobler and Wilson 1990). At any given time, a colony performs different tasks in parallel, with different workers or groups of workers performing these tasks. For a long time, the central question regarding division of labor in eusocial insects concerned how seemingly simple individuals (from a cognitive perspective) can coordinate to perform the necessary tasks in an efficient manner.

Workers of an insect colony are not likely to have a general overview of the state of the colony, nor does a central command exist that distributes workers among tasks. It has been suggested that workers must make choices based on local cues of different behavioral stimuli and information obtained from nestmates (Gordon 1996; Bonabeau et al. 1997; Page and Mitchell 1998). Task specialization and an adequate worker distribution over tasks are not controlled by a central agency but emerge through self-organization from the interactions of workers with their environment and nestmates. Several models have explored various types of behavioral rules that can lead to self-organized division of labor (reviewed in Beshers and Fewell 2001; Johnson 2010). However, these models do not address the question how these rules could arise in the first place (Duarte et al. 2011). Due to the impact of division of labor on colony productivity, and hence fitness, one would expect that the behavioral rules underlying division of labor are targeted by natural selection to produce adequate colony-level responses (Page and Mitchell 1998). Self-organization models tend to neglect the link between division of labor and colony productivity. Contrastingly, models that explicitly analyze the adaptive value of division of labor (Wakano et al. 1998; Wahl 2002; Tannenbaum 2007), tend to neglect the behavioral mechanisms behind it, treating individual task specialization and task generalization as fixed behavioral strategies. There is an urgent need for integrating both approaches and to study the interplay between behavioral mechanisms and evolution (Bonabeau et al. 1997; Page and Mitchell 1998; McNamara and Houston 2009). Few models have attempted to do this (see Waibel et al. 2006; Tarapore et al. 2009). Moreover, these models have not focused specifically on the evolution of specialization and its relationship with colony fitness.

**Fig. 1:**
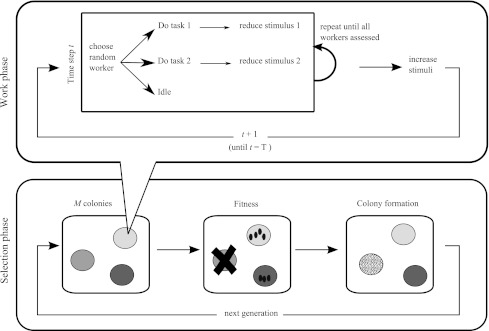
Schematic representation of the model, encompassing the time scale of self-organization (“work phase”) and the time scale of evolution (“selection phase”). At the start of each generation, *M* pairs of reproductives found colonies, each with *N* workers. The colonies go through a work phase where worker behavior is governed by the threshold model of division of labor: depending on whether task-specific stimuli are higher or lower than the genetically determined internal thresholds, workers will perform task 1, task 2, or do nothing. Each task-specific stimulus increases from one time step to the next, and it decreases again whenever a worker performs the task. After *T* time steps, colony fitness is determined as a function of the amount work performed and its distribution over tasks. Colonies produce reproductives proportionally to their fitness, and these individuals found new colonies, which will enter a new work phase

The integration of the perspectives of self-organization and evolution is straightforward to achieve by clearly distinguishing between the timescale of self-organization (within generations) and the timescale of evolution (between generations). Within generations, individuals have a genetic make-up that determines the behavioral rules to which they obey. Division of labor may emerge from the interaction between individuals. Depending on how well the emergent outcome fits colony needs, colonies will achieve lower or higher fitness, i.e., they will produce fewer or more reproductive individuals. Due to selection in the course of the generations, those behavioral rules that lead to adaptive division of labor will thrive (as illustrated by Fig. 1).

In our study, we consider the evolution of self-organized division of labor by introducing evolution in a well-known self-organization model, the response threshold model (Bonabeau et al. 1996; Bonabeau et al. 1998). This model was chosen as point of departure because it has become a reference model for task choice among empiricists (e.g., Detrain and Pasteels 1991; Page et al. 1998; O'Donnell and Foster 2001; Weidenmüller 2004; Robinson et al. 2009). The response threshold model assumes that individuals have inherent thresholds to respond to stimuli associated with specific tasks and, in a group, the individuals with the lowest threshold for a task will perform this task more often. An intuitive analogy in terms of human behavior is the sharing of house chores in humans: people with the lowest threshold for dish-washing, for example, will respond to the smallest accumulation of dishes, and will therefore do the dishes most of the times. Division of labor emerges from the differences between individuals in their thresholds.

Different versions of the response threshold model have looked at the effect of threshold reinforcement, colony size, number of tasks and genetic diversity (Theraulaz et al. 1998; Gautrais et al. 2002; Merkle and Middendorf 2004; Graham et al. 2006; Jeanson et al. 2007; Gove et al. 2009) on division of labor and colony performance. These studies assume that task stimuli are well-mixed in the environment; the cues used by individuals to choose tasks are therefore global. A recent article has explored the effect of spatial distribution of task stimulus on worker activity (Richardson et al. 2011) based on response thresholds; the results of this study suggest that a spatially explicit response threshold model, with local cues, shows similar behavior as a non-spatially explicit model with global cues, at least when only one task is considered. Johnson (2010) showed that when considering multiple tasks, the spatial distribution of task stimuli can lead to short-term specialization, even in the absence of thresholds. We are interested in the evolution of long-term specialization, in which threshold distributions are thought to play a large role. We therefore focus on the simplest version of the response threshold model that allows for division of labor, considering only two tasks and fixed thresholds during worker lifetime (Bonabeau et al. 1996).

The initial model by Bonabeau et al. (1996) assumed from the start that there were two groups of individuals in a colony (referred to as castes), possessing different thresholds for the existing tasks. When differences are assumed a priori it is not surprising to find that individuals behave differently and division of labor emerges. Our main goal is to investigate under which conditions consistent differences in individual thresholds may evolve from a homogeneous population and thus give rise to division of labor. We also test the ability of the response threshold model to generate an adaptive distribution of workers over tasks.

We largely follow previous work on the response threshold model when implementing the dynamics of task-associated signals. If the stimulus for a task is above the threshold value of an individual, the individual has a high probability to perform the task; otherwise, the individual will be less likely to perform the task. Task stimuli decrease with work performed, making it less likely for individuals with higher thresholds to become engaged in the task later on. We start from homogeneous populations (where all individuals have identical thresholds) and allow parameters of the response threshold model to evolve, in order to investigate the conditions under which threshold differentiation (leading to division of labor) may evolve. In this context we also examine how multiple mating affects the evolution of thresholds and division of labor.

We focus on two aspects of division of labor: specialization, i.e., the probability that individuals stick to the same task, and work distribution, i.e., the proportion of workers performing the different tasks. Specialization may be adaptive for two reasons: first, specialists may become more efficient at their task, due to learning, training, or gain of valuable information related to the task; second, specialization may allow the colony to avoid the costs of switching tasks (due to traveling time between task locations or cognitive costs) (Dornhaus 2008). The distribution of workers over tasks is also crucial since it should be adequate to the colony’s needs (Gordon 1996).

## Model structure

### Within-colony dynamics

Individuals are assembled in *M* colonies with a fixed number *N* of workers (unless stated otherwise, *M* = 1,000 and *N* = 100 in our simulations). Within-colony dynamics largely follows Bonabeau et al. (1996) (see sections A and B of the Electronic supplementary material (ESM)). Individuals possess a threshold for each of the two existing tasks (*θ*_*i*_, *i =* 1, 2) that may differ among individuals and is fixed throughout the work phase. At *T* discrete time steps *t* (0 < *t* ≤ *T*, where *T* = 100 in our simulations), we assess all individuals for task choice in a random order. Individuals perceive task-associated stimuli *S*_*i*_ with an error *ε*_*i*_ drawn from a normal distribution of mean 0 and standard deviation *σ* = 1. For a given task, the individual is willing to perform the task if the perceived stimulus is larger or equal to the threshold. Otherwise, the individual will not perform the task. This is summarized by the function:

1 $$ \phi ({S_i},{\theta_i},{\varepsilon_i}) = \left\{ {\matrix{{*{20}{c}} {1,} \hfill & {\text{if}} \hfill & {{S_i} + {\varepsilon_i} \geqslant {\theta_i}} \hfill \\ {0,} \hfill & {\text{if}} \hfill & {{S_i} + {\varepsilon_i} < {\theta_i}} \hfill \\ } } \right. $$

Individuals not motivated to perform any task will stay idle. Individuals motivated to perform one of the tasks will work on the task (Fig. 1). If individuals are motivated to do both tasks, they will perform one of the tasks at random. We assume that when an individual works on a task, the corresponding stimulus is immediately reduced (see below). Hence, different individuals may perceive different levels of stimulus for two reasons: the error (or noise) in stimulus perception and the order in which stimuli are assessed.

In line with the Bonabeau et al. (1996) model, stimuli change in time as follows: there is a constant increase *δ*_*i*_ with every time step and a decrease of *α*_*i*_ with every active worker, where *α*_*i*_ is the efficiency of work (how many work units an individual can do per time step). The stimulus dynamics is therefore described by the equation:

2 $$ {S_i}(t + 1) = {S_i}(t) + {\delta_i} - {\alpha_i} \cdot {A_i}(t) $$

where *A*_*i*_(*t*) is the number of workers active with task *i* at time step *t*. In our simulations, *δ*_*i*_ and *α*_*i*_ have the same values for all individuals and tasks (*δ*_*i*_ = 1 and *α*_*i*_ = 0.03), unless indicated otherwise. These values were chosen because preliminary simulations indicated that these values required the engagement of a majority of workers but were still well within the work capacity of the colonies. The chosen values are also equivalent to the values of stimulus increase used in Bonabeau et al. (1996), thereby rendering comparisons between the models easier.

Stimulus values have a lower boundary at zero; if reduced below zero by a worker, the stimulus is reset immediately to zero. There is no upper boundary to the stimulus level.

### Fitness

For the evolutionary analysis, we considered two scenarios on how colony fitness depends on the work performed. In each scenario, we make assumptions on how colony productivity *w*(*t*) at time step *t* might depend on the work performed on tasks 1 and 2 (*A*_1_(*t*) and *A*_2_(*t*), respectively). Subsequently, we assume that colony fitness *W* is proportional to the geometric mean of these productivity values over time (neglecting the first ten time steps, to avoid initialization effects).

In our standard scenario, we assume that the productivity at a given time unit is given by the weighted geometric mean of *A*_1_(t) and *A*_2_(t):

3 $$ w(t) = {\left[ {{A_1}(t)} \right]^{\beta }} \cdot {\left[ {{A_2}(t)} \right]^{{1 - \beta }}} = A(t) \cdot {p_1}{(t)^{\beta }} \cdot {p_2}{(t)^{{1 - \beta }}} $$

where $A (t) = {A_1 (t)} + {A_2} (t)$ is the total number of acts performed for both tasks and ${p_i} = {{{{A_1 (t)}}}} / A(t) .$ is the proportion of work devoted to task *i*. The exponent *β* is a weighing factor indicating the relative importance of tasks 1 and 2, with 0 < *β* < 1. If *β* = 1/2, then both tasks are of the same importance; if *β* = 3/4, the optimal work distribution is 3:1, with task 1 being performed three times more than task 2. Considering different values of *β* allows us to test the ability of colonies to achieve different adaptive worker distributions. The multiplication of different components forces colonies to work for both tasks; working for only one task results in zero fitness.

As we will see, the above scenario puts much emphasis on the total amount of work done, downplaying the distribution over tasks. To correct for this, we considered another scenario (Eq. 4). We changed Eq. (3) by strongly accentuating the effect of the work proportion *p*_1_:

4 $$ w(t) = A(t) \cdot \exp \left( { - \frac{{{{({p_1}(t) - \beta )}^2}}}{{2{\sigma^2}}}} \right). $$

The second term in Eq. 4 is a Gaussian with maximum in *p*_1_ = *β* that drops rapidly to zero if the “standard deviation” *σ* is small (in our simulations, *σ* = 0.1).

To start a new generation, each colony produces a number of reproductives proportional to the colony’s fitness. *M* pairs of reproductives are drawn at random from the offspring pool to form the new colonies. In the version of the model where colony foundresses are multiply mated (polyandry), *M* foundresses are first chosen at random; for each foundress, *m* mates are then chosen at random. We also considered the situation where both parents mated multiply (polygynandry), but since the results did not differ from polyandry they will not be presented here.

Ten replicate simulations of every parameter combination were run for each model, for 40,000 generations.

### Inheritance

New individuals in a colony originate from the mating of the two parents of the colony, or from the mating of the colony foundress with one of *m* males, in the case of multiple mating. Offspring production occurs at two moments: a fixed number of *N* workers are produced before the start of the working phase, and reproductive offspring is produced at the end of the working phase. In case of multiple matings, paternity is equally shared among males; for each offspring, the father is chosen at random from the males with which the female has mated.

For simplicity, all individuals are haploid and genetically characterized by two genes, encoding the two task thresholds as real numbers, *θ*_1_ ≥ 0 and *θ*_2_ ≥ 0. When a new individual is produced, with probability *r* (the recombination rate) it inherits one threshold from the mother and the other threshold from the father. With probability 1 − *r* the individual inherits both thresholds from the same parent. The recombination rate lies between zero, when the two thresholds are inherited as one gene, and one half, when the thresholds segregate independently. For the simulations shown in the main text, *r* = 1/2. We show results for *r* = 0 in the ESM.

Mutations occur with probability *μ* per gene whenever new offspring is formed. The mutation step size is drawn from a normal distribution of mean zero and standard deviation *σ*_*μ*_. Thresholds must be equal or larger than zero; when a mutation causes thresholds to fall below zero, they are reset to zero. No upper limit is set to the value of thresholds. In order to speed up evolution we chose a high mutation probability (*μ* = 0.1). This choice was compensated by the use of a relatively small mutation step size (*σ*_*μ*_ = 0.1).

### Specialization

When an individual starts a task, we determine whether this is the same or a different task than the one previously done. The probability *q* of performing the same task in two subsequent time steps is a measure of individual specialization. We average *q* over all workers in a colony, and normalize the mean $\bar{q}$ by dividing it by the probability that individuals would stay in the same task due to chance alone. This probability is given by $p_1^2 + p_2^2$, where *p*_1_ and *p*_2_ correspond to the colony’s proportion of acts for task 1 and 2, respectively. Note that individuals that remained idle for the entire simulation are not taken into account for the calculation of $\bar{q}$. It can be shown that $0 < \bar{q} /(p^{2}_{1}  + p^{2}_{2} ) \leqslant 2$. To obtain a standardized measure for the degree of mean worker specialization, we subtract 1 to obtain:

5 $$ D = \frac{{\bar{q}}}{{p_1^2 + p_2^2}} - 1 $$

Hence, *D* varies from −1 to 1. For *D* = 0, $\bar{q} = {p_1}^2 + p_2^2$, implying that, individuals choose tasks randomly. *D* = 1 is achieved if all individuals fully specialize on one task, thus dividing labor. *D* = –1 indicates that individuals always switch between tasks from one time step to the next. In our simulations, *D* was always larger than or equal to zero.

## Results

### Evolution of thresholds and the work distribution

**Fig. 2:**
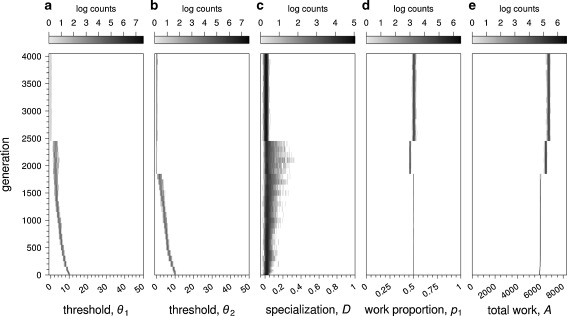
Evolutionary simulation of the response threshold model, for *β* = 0.75, under fitness scenario (Eq. 3). Frequency distributions of thresholds (**a**, **b**), specialization (**c**), work proportion (**d**), and the total amount of work performed (**e**) over evolutionary time are shown. Thresholds for both tasks were initialized at ten for all individuals. The graphs show the first 4,000 generations. Grey scales on top indicate the frequency distribution of the trait depicted over *M* colonies (*M* = 1,000). Within about 2,000 generations, the two thresholds *θ*_1_ and *θ*_2_ evolve to values close to zero (threshold values of each colony’s parents are shown). Throughout the simulations, the proportion of work spent on task 1 (*p*_1_; **d**), was close to 0.5, although a work distribution of ${\hat{p}_1} = \beta = 0.75$ would have been optimal. Specialization *D* first increased and later dropped to zero again. The total amount of work (*A* = *A*_1_ + *A*_2_) increased over evolutionary time

Figure 2 shows a representative simulation for the standard fitness scenario (Eq. 3) and *β* = 0.75. All replicates showed essentially the same behavior. In less than 4,000 generations, both thresholds evolved to zero levels (Fig. 2a, b). At first, a certain degree of specialization evolved in some colonies (*D* varying between 0 and 0.5), but specialization disappeared as soon as both thresholds reached zero (Fig. 2c). Throughout evolutionary time, the evolved value of *p*_1_ was always close to 0.5 (Fig. 2d). Hence, both tasks were performed equally often, even though a value of ${\hat{p}_1} = \beta = 0.75$ would have been optimal. In view of the drop in threshold values, it is not surprising that the total amount *A* of work performed increased in the course of evolutionary time (Fig. 2e).

In many other simulations (see below) we also found that selection in favor of a biased work distribution is not very efficient and that the work distribution tends to stay close to a value of *p*_1_ = 0.5. To understand this, we took a closer look at the self-organization part of the model, namely at the stimulus dynamics (Eq. 2). At equilibrium (*∆S*_*i*_ = 0), the number of workers for task *i* is:

6 $$ {\hat{A}_{{ i}}} = {{{{\delta_i}}} \left/ {{{\alpha_i}}} \right.} $$

Therefore, at stimulus equilibrium, the number of workers in each task depends solely on the stimulus parameters and not on the threshold values (see section B of the ESM). As long as the values of *δ*_*i*_ and *α*_*i*_ are the same for all tasks (as in our simulations), ${\hat{A}_i}$will take the same value for all tasks *i*. Hence, *any* value of the thresholds should result in the same (unbiased) distribution of workers over tasks, given that stimulus equilibrium can be reached. Note, however, that the threshold values might affect whether and after how many time steps a stimulus equilibrium is reached in a simulation. These considerations are corroborated by (non-evolutionary) simulations of the work phase of the response threshold model (see Fig. S6 of the ESM).

The fact that the thresholds evolve to zero (Fig. 2a, b) is perhaps the most remarkable result of the simulations. Once the thresholds have disappeared, the whole threshold mechanism breaks down. Individuals are *always* motivated to perform any of the tasks, and individuals do not differ in their task preference.

In the simulations considered thus far, the thresholds presumable converged to zero because this maximizes the total amount work a colony can do. In fact, all individuals are busy all of the time when their thresholds are equal to zero. An obvious reason for this outcome might be the choice of fitness scenario (Eq. 3), giving a high premium to an increase in *A*. For this reason, we also considered the alternative fitness scenario (Eq. 4).

**Fig. 3:**
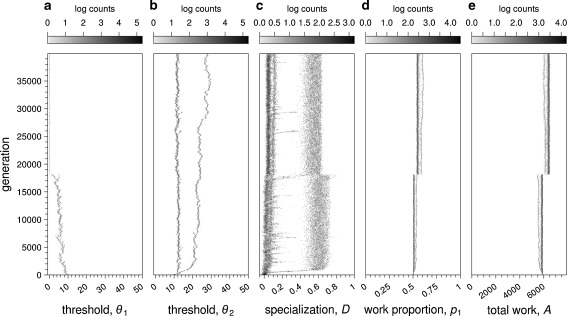
Evolutionary simulation of the response threshold model, for *β* = 0.75, under fitness scenario (Eq. 4) (*σ* = 0.1). Graphical conventions follow Fig. 2. *M* = 100. *θ*_1_ decreases to zero (**a**) and *θ*_2_ branches (**b**). A proportion of colonies shows worker specialization, with *D* values larger than 0.5 (**c**). Coinciding with the drop of *θ*_1_ to zero, work proportion, *p*_1_ increases slightly but does not reach the optimal value of 0.75 (**d**). Total amount of work, *A*, also increase over evolutionary time (**e**)

The evolutionary outcome changed when selection on a biased work distribution was made very strong, as in fitness scenario Eq. (4). Figure 3 shows the outcome for parameter values *β* = 0.75 and *σ* = 0.1. Now, the threshold *θ*_1_ for the “favored” task 1 still converged to zero, but the threshold for the other task, *θ*_2_, exhibited evolutionary branching (Geritz et al. 1998) (Fig. 3a, b). In other words, a polymorphic population results where part of the population has *θ*_2_ values close to 10, while the rest of the population has higher values that increase to values around 25. Due to branching, *p*_1_ evolved to a higher value than 0.5 but still it remained at a considerably lower level than the optimal value ${\hat{p}_1} = \beta = 0.75$ (Fig. 3d). Averaging across replicates, 33.8 ± 3.8 % (mean ± SD) of colonies show a high degree of specialization (*D* ranging between 0.5 and 0.7; Fig. 3c). Specialization arises in those colonies where the parents differ in their values at the *θ*_2_ locus (Fig. S5 of the ESM).

Evolution of division of labor under these circumstances is a side-effect of selection on work ratio. Increasing thresholds leads to colonies achieving stimulus equilibrium later during the simulation; hence, overall, less work will be done for the task with the highest thresholds. Colonies which are polymorphic for *θ*_2_ have an advantage because workers with *θ*_2_ = 10 will maintain the stimulus at a level between 15 and 20 (results not shown), hence below *θ*_2_ = 25. Consequently, in monomorphic colonies, fewer workers are willing to perform task 2 than in polymorphic colonies.

### Evolution of specialization

In the previous simulations specialization only evolved when a bimodal distribution of thresholds evolved due to evolutionary branching (as in Fig. 3b). This indicates that the distribution of thresholds is key to the emergence of specialization. Indeed, a bimodal distribution of thresholds is assumed from the start in the self-organization model of Bonabeau et al. (1996). This model considers two “castes” of workers (“majors” and “minors”) such that ${\theta_1}^{\text{majors}} > {\theta_1}^{\text{minors}}$ and ${\theta_2}^{\text{majors}} < {\theta_2}^{\text{minors}}$. Not too surprisingly, the minors specialize on task 1 while the majors specialize on task 2. We obtained the same result under less constrictive conditions when initializing a population by drawing individual thresholds from two bivariate normal distributions (Fig. S6b in the ESM). As in the model of Bonabeau et al. (1996), task specialization converged to the maximal level *D* = 1.

The question therefore arises when such a distribution of thresholds can evolve from scratch, starting from a homogeneous population. In Fig. 3, the diversification of threshold *θ*_2_ was driven by strong selection for a biased work distribution. To exclude this effect, we will from now on assume *β* = 0.5. Moreover, we will mainly focus on our standard fitness scenario (Eq. 3); in the ESM, we briefly present the results for fitness scenario (Eq. 4).

**Fig. 4:**
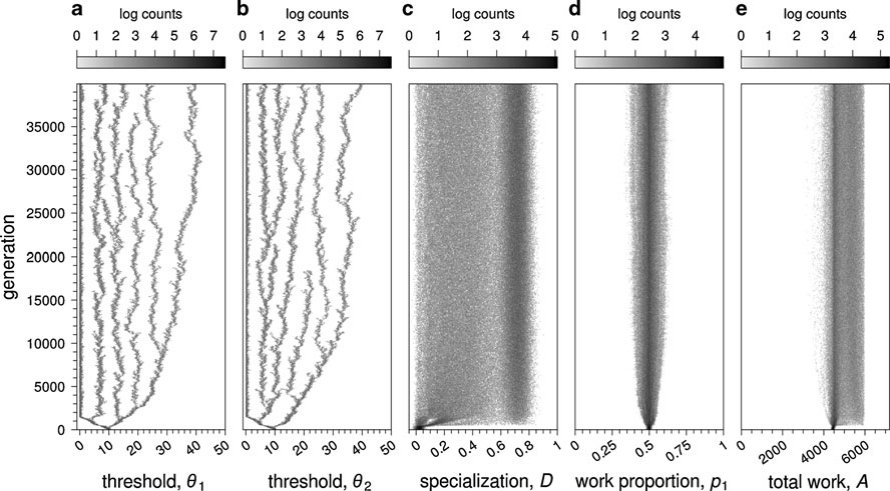
Evolutionary simulation of the response threshold model, when switching tasks is costly (*c* = 2) and *β* = 0.5, under the standard fitness scenario (Eq. 3). The setup of the simulations and the graphical conventions are identical to Fig. 2. Both thresholds diverged quickly into equally spaced multiple branches (**a**, **b**). Worker specialization increased quickly in the first 500 generations, with 55.6 ± 2.8 % (mean ± SD) of the colonies having a *D* value larger than 0.5 (**c**). The work distribution varied among colonies around *p*_1_ = 0.5 (**d**). In the first generations, colonies have perform a low amount of work (**e**), reflecting the fact that workers switching tasks have to stay idle for *c* = 2 time periods. Part of the population recovers from this cost by evolving specialization

In order to investigate whether evolution can shape colonies with a high degree of task specialization, we consider a scenario where worker specialization has a direct positive effect on colony fitness. To this end, we assume that switching between tasks is costly in terms of time. This is a simple, mechanistic cost that may result from the fact that tasks are spatially distributed (Sendova-Franks and Franks 1995), without having to make assumptions on the cognitive aspects of task performance. In our model, the switching cost is a time delay: individuals switching tasks must wait *c* time steps before engaging in the new task. We investigated values of *c* ranging from one to eight time steps). Evolution of worker specialization occurred for *c* ≥ 2, in a highly consistent way across replicates. In all replicate simulations, the increase in specialization was associated with multiple evolutionary branching of the two thresholds in the population. A typical simulation is shown in Fig. 4. Some colonies achieved a high degree of specialization, but there was much variation in specialization across colonies (Fig. 4c). This outcome was again highly consistent across simulations (Fig. S12a in the ESM). Averaging across replicate simulations, 55.6 ± 2.8 % (mean ± SD) of the colonies showed a value of *D* > 0.5 at the end of 40,000 generations. As a result of specialization, individuals are able to avoid the cost of switching and the number of working periods increases (Fig. 4e). Across colonies, there is a clear positive relationship between specialization and the number of working periods achieved (Fig. S8 in the ESM).

The level of specialization of the colonies depends on the thresholds possessed by the parents of the colony. When parental thresholds are similar for both tasks (i.e., ${\theta_1}^{\text{mother}} = {\theta_1}^{\text{father}}$and ${\theta_2}^{\text{mother}} = {\theta_2}^{\text{father}}$), colonies show low mean specialization due to high similarity of thresholds among workers (Fig. S9 in the ESM). When parental thresholds differ for both tasks, colonies show higher mean specialization. Yet, notably, maximal specialization (*D* = 1) is never reached, even when switching costs are very high (results not shown). This can be understood by considering an example: a colony where parental thresholds are ${\theta_1}^{\text{mother}} = {\theta_2}^{\text{mother}} = 10$ and ${\theta_1}^{\text{father}} = {\theta_2}^{\text{father}} = 40$. Owing to recombination, workers produced in this colony will fall into 4 types according to their thresholds: *θ*_1_ = *θ*_2_ = 10; *θ*_1_ = *θ*_2_ = 40; *θ*_1_ = 10 and *θ*_2_ = 40; and *θ*_1_ = 40 and *θ*_2_ = 10. The first two types of workers will have no preference for either task, the third type will be more likely to perform task 1 and the fourth type will be more likely to perform task 2. Hence, the mean level of specialization in the colony is decreased by the presence of workers of types 1 and 2.

The work distribution was also variable across colonies (Fig. 4d), in a consistent way across simulations (Fig. S10b in the ESM). Averaging across simulations, 73.3 ± 2.2 % (mean ± SD) of the colonies show a proportion of work for task 1 between 0.45 and 0.55 (Fig. 4d).

Under strong selection on worker distribution (fitness scenario (Eq. 4)), specialization typically did not evolve when *β* = 0.5, for any of the switching costs tested (see Fig. S13 in the ESM).

### Effects of multiple mating

**Fig. 5:**
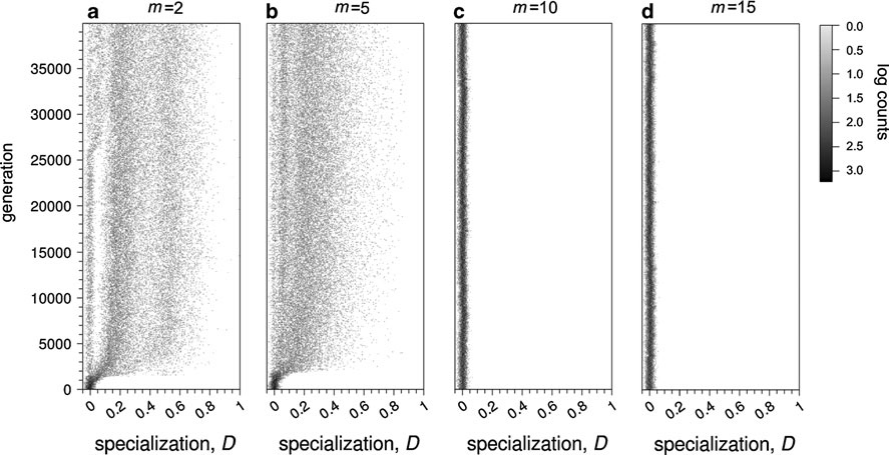
Evolution of worker specialization, *D*, in example simulations with different number of matings, *m*, under the standard fitness scenario (Eq. 3). The evolved level of differentiation decreases with the number of matings

It is often assumed that multiple mating of the queens has a beneficial effect on division of labor in social insect colonies (Oldroyd and Fewell 2007). To investigate whether this effect also occurs in the threshold model, we allowed foundresses to mate *m* = 2, 5, 10, or 15 times. As shown in Fig. 5, the number of matings does indeed have a strong effect on the evolution of specialization. Perhaps surprisingly, however, the evolved degree of specialization decreased with the number of matings. When females mated with two different males, the simulation results resemble those in the monogamy scenario considered in “Evolution of specialization.” However, fewer branches of the thresholds evolved within the runtime of a simulation (see example simulation in Fig. S14 in the ESM), and a lower proportion of colonies achieved *D* > 0.5 (Fig. 5a). When increasing the number of matings, threshold branching and the associated evolution of specialization occurred in fewer and fewer simulations. For *m* = 5, 10, and 15 matings, the number of simulations in which the thresholds branched within 40,000 generations was eight, five, and two out of ten replicates, respectively.

## Discussion

In our study, we have analyzed the response threshold model (Bonabeau et al. 1996) from an evolutionary perspective. Previous work considered colonies with a priori differentiated castes, where it is not too surprising that division of labor will emerge. Our study shows that evolution, starting from fully undifferentiated, unspecialized workers, can lead to a state where differentiated workers divide labor in a self-organized manner. The trajectory to specialization involves evolutionary branching (Geritz et al. 1998) at the loci influencing task choice. Evolution of division of labor occurred when task-switching incurred costs to the colony, in terms of time that individuals had to spend inactive when transitioning between tasks. Interestingly, task specialization also evolved without direct benefits of specialization when task-related thresholds branched for other reasons (strong selection in favor of a biased distribution of workers over tasks).

Our results highlight an important drawback of the behavioral architecture encapsulated in the response threshold model. We have shown that, at stimulus equilibrium, the distribution of workers over tasks is not governed by the distribution of thresholds in the population, but by the parameters of the stimulus dynamics. Even when selection on a particular distribution of workers over tasks is strong, the response threshold mechanism does not easily evolve a worker distribution that differs from the requirements imposed by the stimulus dynamics parameters. This constraint is relevant for the course and outcome of evolution. While it is easy to imagine how internal properties like thresholds could differentiate in the course of evolution, this is much less so for the properties of the stimulus dynamics. At least for tasks in which the stimulus corresponds to environmental cues such as temperature, humidity or food availability, the stimulus dynamics will be mainly externally imposed and hence, not subject to evolution. This general property of the threshold model indicates how the model could be put to the test experimentally. Our analysis leads to two specific empirical predictions. First, when placed under the same stimulus conditions, colonies with different distributions of thresholds should eventually produce the same distribution of workers over tasks. Second, when placed under different stimulus conditions, colonies with the same threshold distribution should, in a predictable manner, achieve different distributions of workers over tasks.

In our standard scenario, specialization only evolved under relatively high switching costs (i.e., when switching costs lead to a 25 % or larger decrease in colony performance; Fig. S7 in the ESM). When costs were lower, the evolutionary tendency toward low thresholds was dominant, hampering evolutionary branching. In nature, the costs involved in switching tasks are most likely dependent on the tasks considered. It is plausible to consider that high costs, time-wise, are involved in switching between tasks like foraging and nursing, since the physical location of these tasks is far apart. Furthermore, to switch between such tasks, individuals may also “pay” physiological costs—for example, in the honey bee, the transition to foraging implies physiological changes which take some time to reverse (Huang and Robinson 1996). However, for other tasks which are closely located and/or physiologically independent, such as nursing and maintenance of nest temperature, it is not plausible to consider high switching costs and we would expect switching to occur more often between these tasks, as seen in middle-aged honey bees (Johnson 2003).

The main constraint on specialization in the current implementation of the threshold model is the need for non-random variation, where a part of the colony must have *θ*_1_ >> *θ*_2_, and the other part *θ*_1_ << *θ*_2_. Note that here division of labor is an emergent property, since it results from the interaction of individuals with different combinations of thresholds. The fact that threshold values in the population evolve into multiple branches (i.e., the population is polymorphic for threshold values) decreases the probability that individuals inherit similar threshold values, thus helping in creating the diversity needed for specialization.

Such a constraint implies that the optimal colony phenotype is destroyed by recombination. In accordance with this argument, we observed higher values of specialization in simulations where thresholds evolved in complete linkage (see section E of the ESM). Yet, even in the absence of recombination, a part of the colonies showed no specialization, owing to the pairing of individuals with non-complementary thresholds. In natural systems, the lack of division of labor resulting from unfavorable combinations of parents could be avoided through the evolution of disassortative mating. If mating would preferentially occur between reproductives with a complementary threshold, a much higher degree of task specialization within the colony would result. Unfortunately, little is known about mate choice in social insects and it seems that it is unlikely to be an important force in species such as ants and termites where males cannot mate multiply (Boomsma et al. 2005).

There is some evidence that multiple mating has beneficial effects on division of labor owing to genetic task determination (e.g., Oldroyd and Fewell 2007). Improved colony performance and increased disease resistance due to high intra-colony genetic diversity are the two major explanations for the presence of multiple mating in several species of eusocial insects (Brown and Schmid-Hempel 2003). A few theoretical studies, based on the response threshold model, have supported the hypothesis that multiple mating does have beneficial effects on colony performance (Graham et al. 2006; Gove et al. 2009; Tarapore et al. 2009). In view of this evidence, our finding that increased number of matings did not facilitate the evolution of specialization is surprising. A possible explanation is that mutations in threshold values do not have as strong an effect on colony fitness if the foundress is multiply-mated. Under single-mating, a male and female with thresholds varying in the opposite direction (i.e., at the extremes of the threshold distribution), would produce a colony with considerably higher fitness, thus leading to a quick spread of the new alleles in the population. If the female is multiply-mated, parentage of workers will be shared equally among males (the majority of which not carrying the beneficial mutations) and only a small proportion of workers within the colony will possess the threshold combination leading to specialization. Our results suggest that multiple mating may only promote specialization if genetic diversity in task-choice alleles is already present. Our findings are also in line with previous work that showed that, in general, multiple mating decreases the variance in colony performance, and therefore is less beneficial when the average colony performance is poor (Rueppell et al. 2008). In our model, average colony performance can be considered poor when task switching is costly and colonies are monomorphic. These results once again illustrate how adding evolution to self-organization models may lead to different insights and conclusions.

Colony size has been argued to influence division of labor, with larger colonies having more specialized workers (Anderson and McShea 2001). Previous work on threshold models supports this argument (Gautrais et al. 2002; Merkle and Middendorf 2004; Jeanson et al. 2007). In our study, we focused on a colony size of 100 individuals, but we also considered colonies consisting of 20, 50, 500, and 1,000 individuals. Colony size did not qualitatively affect the results obtained for any of the studied colony traits (see Fig. S17 in the ESM). The discrepancy between previous models and ours is likely owing to differences in the implementation of the stimulus dynamics and of the threshold mechanism itself. A more technical comparison of different threshold models would be useful to fully understand how colony size can influence division of labor under a threshold mechanism.

In evolutionary models of division of labor, choosing an adequate measure of colony productivity or fitness can be rather complex. In real social insects, the actual relationship between workload completed, ratio of work over tasks, and colony fitness is not well defined. Here we tested two functions which give emphasis to different fitness components. Using a fitness function that gives high priority to the distribution of workers over tasks produced different results than our standard fitness scenario. Even in the absence of switching costs, evolutionary branching of (one of the) thresholds and worker specialization evolved. However, even under these circumstances, the limitations of the threshold model remained and the optimal work ratio was not achieved. Interestingly, the same fitness scenario (Eq. 5) that induced task specialization in the absence of switching costs (for the case *β* = 0.75, favoring a biased distribution of workers over tasks) prevented task specialization even in case of high switching costs when a 1:1 work distribution was optimal (*β* = 0.5). This is likely because branching of thresholds introduces variation in the work distribution, and colonies that deviate from the optimal 1:1 work ratio are severely punished. Hence, in contrast to the other fitness function, the selective pressure on work distribution functions as an obstacle to the evolution of specialization. This illustrates that the simple architecture encapsulated in the threshold model cannot cope optimally with multiple selective pressures.

Several other avenues of research would be fruitful for future studies. One possibility is to consider more open behavioral architectures, for example using evolvable neural networks, where external stimuli are picked up and further processed by various layers of neurons, which eventually determine what kind of behavior results from the given input. Recent studies using neural networks indicate that a diversity of evolutionary outcomes is conceivable under a more open architecture, some of which are impossible in the fixed response threshold model, such as the possibility for the stimulus of one task to influence directly the behavioral output for another task (Lichocki et al. 2012; Duarte et al. in press). Such an open architecture can overcome the constraint of the threshold model that only specific worker distributions over tasks are feasible.

Another avenue of research is to consider the role of phenotypic plasticity as a source of differentiation among workers. Here, we consider behavior to be entirely determined by genetic factors, but in reality it has been found that developmental plasticity plays an important role in generating inter-individual variation (Oster and Wilson 1978; Robinson 1992; Weidenmüller et al. 2009). Likewise, experience has also been shown to have an effect on task choice in real colonies (Ravary et al. 2007). In an evolutionary version of the reinforced threshold model, where thresholds change after task performance, we observe that experience-based specialization overcomes the limitations imposed by recombination and random mating (Duarte et al., in preparation).

Our study is one of the first in a framework where complex adaptive systems are seen as the result of the interplay of natural selection and self-organization. More such studies are needed to help clarify the roles of these two forces in shaping such systems.

## Electronic supplementary material

Below is the link to the electronic supplementary material.

ESM 1 (DOC 2230 kb)
