# Supplementary material for: Evolution of self-organized division of labor in a response threshold model
Source: Behav Ecol Sociobiol. 2012 Mar 22;66(6):947–57. doi: 10.1007/s00265-012-1343-2 (PMC3353103; doi:10.1007/s00265-012-1343-2)
Supplement: Supplementary file 1 — (DOC 2230 kb) [file 265_2012_1343_MOESM1_ESM.doc]

# **Evolution of self-organized division of labor in a response threshold model**

Behavioral Ecology and Sociobiology

Ana Duarte*, Ido Pen, Laurent Keller & Franz J. Weissing

* Corresponding author

Department of Theoretical Biology, Centre for Ecological and Evolutionary Studies, University of Groningen, P.O. Box 11103, 9700 CC Groningen, Netherlands

Current address: Department of Zoology, University of Cambridge, Downing Street, CB2 3EJ Cambridge, United Kingdom

Email: [al638@cam.ac.uk](mailto:al638@cam.ac.uk)

Telephone: +44(0) 1223 334 466

Fax: +44 (0)1223 336676

**Electronic Supplementary Material**

In this supplement, we provide additional information about our model and the dependence of the model behavior on the various model variants and model parameters. The supplement consists of the following six sections:

A. Implementation of the response threshold model

B. Stimulus and worker dynamics in the response threshold model

C. Additional results for the model without switching costs

D. Additional results for the model with switching costs

E. Effect of recombination

F. Effect of colony size

**A. Implementation of the response threshold model**

Our implementation of the response threshold model differs from the original model by Bonabeau *et al.* (1996) in a few aspects. First, we assume more explicitly than Bonabeau and colleagues that stimuli are perceived with noise (see section B). Second, in the original model individuals meet only one of the task stimuli (with equal probabilities); therefore there is never a situation where an individual is willing to do both tasks. Our tie-breaking mechanism in such situations (random choice between the two tasks) is nevertheless comparable to the original implementation. The third difference regards the update of the stimulus with every individual’s action. This implies that individuals may perceive different stimuli, which does not occur in the original implementation. The fourth and last difference is that we waiver the assumption of the original model that, once they have chosen a task, individuals stick to this task for 5 time steps on average, regardless of stimuli values. Hence, at least in part, specialization is already built into Bonabeau *et al.*’s model. In our model, individuals assess task stimuli at every time step.

**B. Stimulus and worker dynamics in the response threshold model**

Bonabeau *et al.* (1996) assumed that – given a stimulus $S_{i}$ and a threshold $\theta_{i}$ – an individual will perform the task with probability

$\Phi_{i}=\frac{S_{i}^{n}}{S_{i}^{n}+\theta_{i}^{n}}$. (S.1)

For large values of the parameter *n*, the “responsiveness” $\Phi_{i}$ comes close to the usual interpretation of a threshold process: $\Phi_{i}=1$ if $S_{i}>\theta_{i}$ and $\Phi_{i}=0$ otherwise. For smaller values of $n$, $\Phi_{i}$ is an S-shaped function of $S_{i}$ (Fig. S1a). The interpretation of (S.1) is that there is always a probabilistic element in decision making.

In our model, we make this probabilistic element explicit, by assuming that stimuli are perceived with a certain error. The probability $\Phi_{i}$ that a given individual with threshold $\theta_{i}$ for task *i* is responsive to the stimulus value *S_i_*, is the expected value of the function , which is defined by eq. (1) in the main text. $\Phi_{i}$ corresponds to the probability that the normally distributed error term $\varepsilon_{i}$ (with mean 0 and standard deviation $\sigma$) is larger than $\theta_{i}-S_{i}$. This probability can be written in the form

$\Phi_{i}=\frac{1}{2}\left[1+\mathrm{erf}\left(\frac{S_{i}-\theta_{i}}{\sigma\sqrt{2}}\right)\right]$ (S.2),

where erf is the Gaussian error function (Abramowitz & Stegun 1972).

As for Bonabeau’s function (S.1), also in our model the propensity of individuals to engage in a task is increasing with the stimulus value in a sigmoidal fashion (Fig. S1b). The steepness of the function in the vicinity of $\theta_{i}$ is increasing with the inverse of $\sigma$, while it is increasing with *n* in Bonabeau’s model. Qualitatively the two approaches give similar results (Fig. S1). Yet, our approach has a clear mechanistic underpinning which lacks in the original response threshold model.


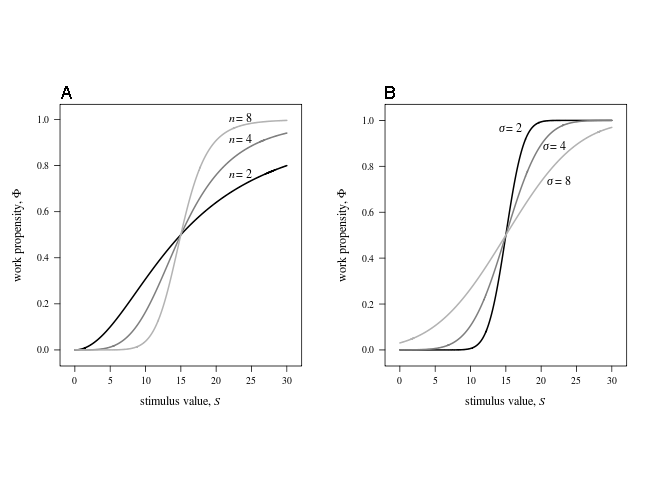


**Fig. S1** Probability $\Phi_{i}$ of an individual with threshold $\theta_{i}=15$ to be responsive to a given stimulus value in (A) the model of Bonabeau *et al.* (1996) and (B) our more mechanistic model

We can now calculate the propensity of an individual with threshold $\theta_{i}$ to engage in task *i* at equilibrium. An individual will take on task *i* (rather than the alternative task *j*) under two conditions: either only task *i* is activated while task *j* is not (probability $\Phi_{i}(1-\Phi_{j})$), or both tasks are activated (probability $\Phi_{i}\Phi_{j}$) and task *i* is chosen at random with probability ½. Accordingly, the expected number of workers engaged in task *i* is given by:

$A_{i}=N\left(\Phi_{i}(1-\Phi_{j})+\frac{1}{2}\Phi_{i}\Phi_{j}\right)$ (S.3)

From eq. (6) in the main text we know that at equilibrium . Therefore, the propensities for individuals to engage in tasks at equilibrium are also the same ($\hat{\Phi}_{1}=\hat{\Phi}_{2}=\hat{\Phi}$) and implicitly given by the equation:

$N\left(\hat{\Phi}(1-\hat{\Phi})+\frac{1}{2}\hat{\Phi}^{2}\right)=\frac{\delta}{\alpha}$ (S.4)

Solving this quadratic equation results in:

$\hat{\Phi}=1-\sqrt{ 1-\frac{2\delta}{\alpha N}}$. (S.5)

Hence, the propensity for a worker to engage in a task at stimulus equilibrium is only dependent on “external” parameters and not dependent on the thresholds of the worker.

**C. Additional results for the model without switching costs**

For the simulations depicted in Fig. 2 of the main text (where $\beta=0.75$) the realized work distribution was close to 1:1, whereas a 3:1 ratio between tasks 1 and 2 would have been optimal. The thresholds evolved to values close to zero, most probably because this increases fitness by minimizing the number of idle individuals. Fitness values could, however, not achieve their maximum levels, due to the inability of colonies to reach the optimal work distribution (Fig. S2).

**
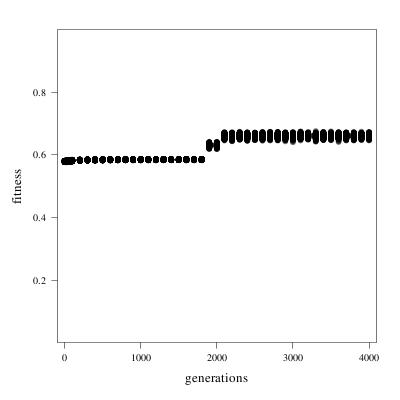
**

**Fig. S2** Relative fitness (i.e., fitness divided by the maximum possible fitness) for each colony in a simulation corresponding to Fig. 2 ($\beta=0.75$).

When thresholds are close to zero, more workers are active than the number at stimulus equilibrium ($\hat{A}_{ i}=\delta_{i}/\alpha_{i}$) would suggest (Fig. S3). This occurs because even at very low stimulus levels many workers will nevertheless be willing to perform the task due to errors in their perception. When thresholds are larger than zero, the equilibrium value of workers is well characterized by eq. (6). We corroborated the analytical result with simulations of the work phase of the response threshold model, without evolution of the thresholds. Simulations were run for colonies with normally distributed thresholds for the two tasks around means ranging from 5 to 30 and standard deviation 1. The ratio $\delta/\alpha$for task 1 was systematically varied, while keeping $\delta/\alpha$for task 2 fixed ($\delta_{1}/\alpha_{1}$ranged from 10 to 50 and $\delta_{2}/\alpha_{2}$ was kept at 10). As expected, the ratio of work done for task 1 and work done for task 2 ($\hat{A}_{1}/\hat{A}_{2}$) at stimulus equilibrium depended only on the ratio $\delta_{1}/\alpha_{1}$, and not on the threshold values ( Fig.S4).

**
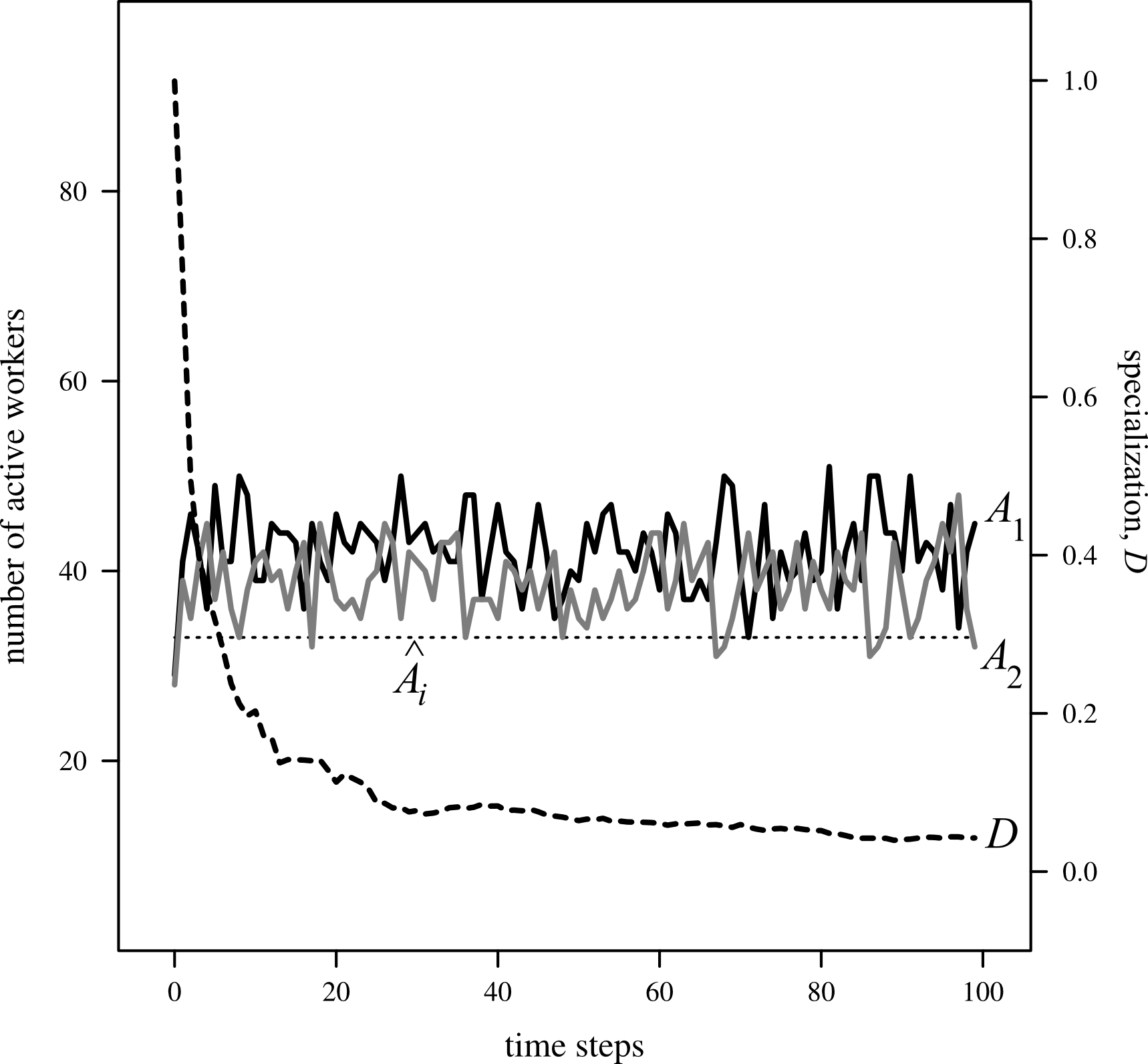
**

**Fig. S3** Worker dynamics in a colony where thresholds have evolved to values close to zero. The number of workers active on task 1 (solid black line) and task 2 (solid grey line) is considerably larger than the values of $A_{ i}$ at stimulus equilibrium (dotted line). During the work phase, worker specialization drops to low values (dashed line)

**
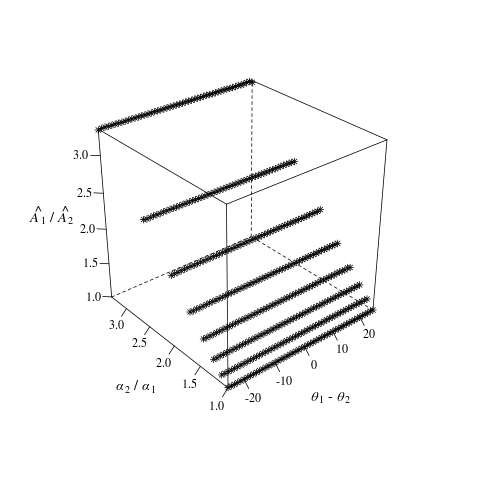
**

**Fig. S4** Ratio of workers for the two tasks at stimulus equilibrium is plotted against different ratios of work efficiency and differences between thresholds. Colonies were initialized from a normal distribution, with the same mean threshold and standard deviation 1. Mean threshold values were varied from 5 to 30, in steps of 1, and every combination was tested. Each point corresponds to the average of 10 simulations. The ratio of workers clearly changes with the ratio of the efficiency parameters, but is not affected by the mean difference between thresholds

In the simulations ran for fitness scenario (4), where there is strong selection on worker distribution, we found that, if a biased distribution was favored, evolutionary branching occurred in $\theta_{2}$and specialization evolved (see Fig. 3 in main text). In Fig. S5 we show the relationship between parental $\theta_{2}$and the level of specialization achieved by the colonies at the end of 40000 generations. Colonies whose parents differ markedly in $\theta_{2}$show higher levels of specialization.


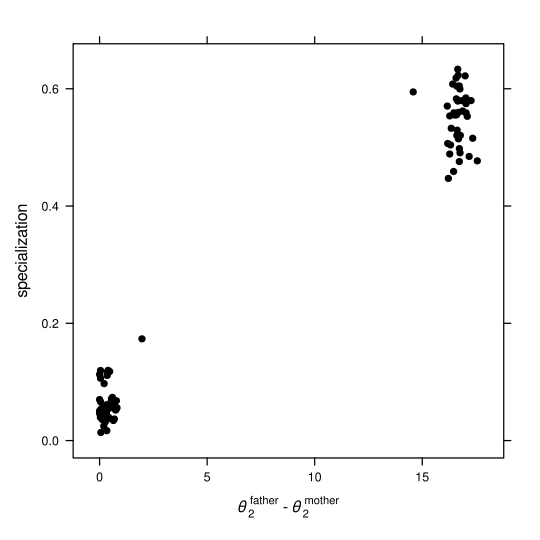


**Fig. S5** Mean worker specialization plotted against the absolute difference between the values of the branching threshold $\theta_{2}$of the colonies’ parents. Each data point represents one colony (*M* = 100) in a representative simulation, after 40000 generations of selection under fitness scenario (4), *β *= 0.75, $\sigma=0.1$.

**D. Additional results for the model with switching costs**

First we ran simulations of the work phase of the threshold model to understand what threshold distributions could bring about specialization. Fig. S6 shows a representative simulation for a case where the thresholds of all colony members are drawn from a unimodal distribution around an (arbitrary) mean for threshold 1 (here $\bar{\theta}_{1}=5$) and a unimodal distribution around a mean for threshold 2 (here $\bar{\theta}_{2}=20$). Irrespective of the considerable difference between threshold values, $A_{1}$ and $A_{2}$ converge to the same level, hence yielding an unbiased work distribution ($p_{1}=0.5$).Mean specialization is low, indicating that workers switch randomly between tasks.

When initializing a population by drawing individual thresholds from two bivariate normal distributions (Fig. S6B), one with $\bar{\theta}_{1}=5$and $\bar{\theta}_{2}=20$ and the other $\bar{\theta}_{1}=20$and $\bar{\theta}_{2}=5$. As in the model of Bonabeau *et al*. (1996), task specialization converged to the maximal level $D=1$. A high degree of specialization can therefore be achieved if the thresholds in individual workers are negatively related to each other (as in Fig. S6B).


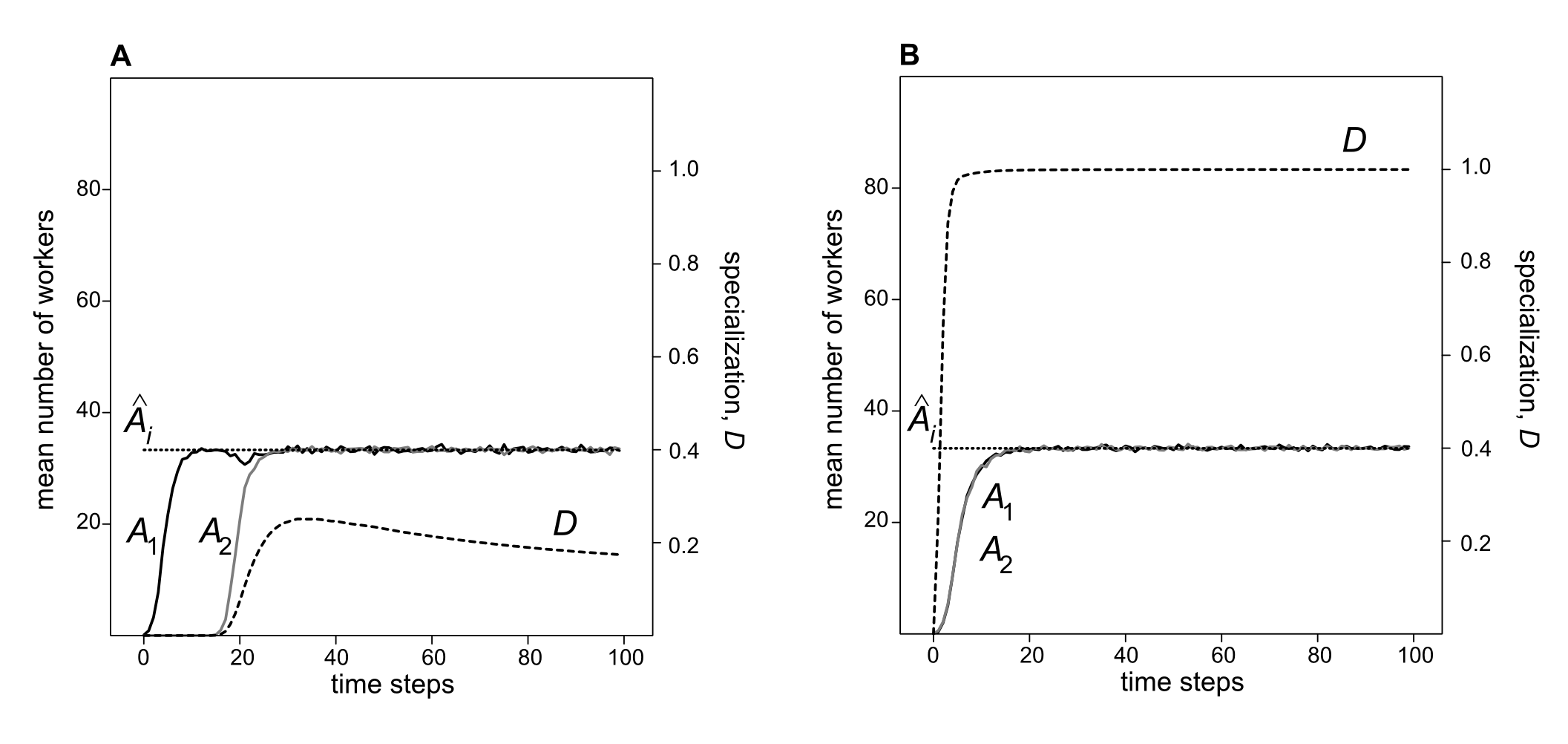


**Fig. S6** Distribution of workers over tasks and degree of specialization for a given distribution of thresholds in a colony. Both panels show the average of 100 colonies. In (A) the two thresholds of an individual were drawn independently from two normal distributions with mean $\theta_{1}=5$ and mean $\theta_{2}=20$ and standard deviation = 1. In (B) they were drawn from two bivariate normal distributions with a negative correlation -0.5: for half of the workers the bivariate normal distribution had means $\theta_{1}=5$ and $\theta_{2}=20$, while for the other half the distribution had means $\theta_{1}=20$ and $\theta_{2}=5$. In both panels, the number of active workers per task converge to the equilibrium value (eq. (5); dotted line). In panel A), specialization $D$ (dashed line) only reaches a low level, while $D$ converges to the maximal value 1 in panel B)

In order to investigate if specialization could evolve from scratch in the response threshold model, we added a cost to switching tasks, in the form of a delay in task performance whenever individuals choose to switch from their previous task. For costs equal or larger than 2 time steps, the number of acts contributing for fitness decreased by one fourth (Fig. S7). Evolution of specialization occurred through evolutionary branching of the thresholds, for *c* ≥ 2. This was associated with an increase in work performed, which is brought back to values close to the work performed incolonies not suffering from switching costs, showing that specialized colonies are able to avoid switching and therefore spend more time working (Fig. S8).


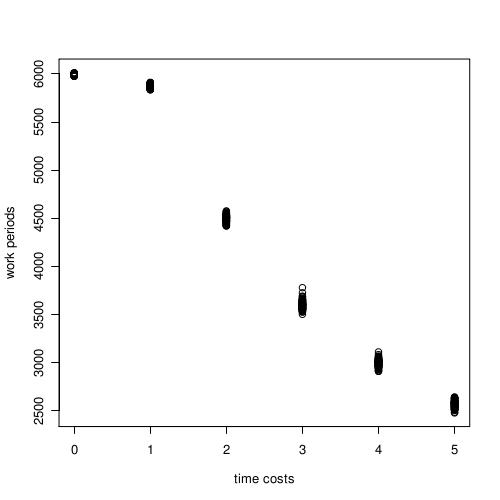


**Fig. S7** Effect of costs of task switching on the total amount of work performed. Six populations, with 100 colonies each, were simulated under different costs of task switching. Thresholds did not evolve but were randomly assigned to individuals, according to a normal distribution (mean = 5, sd = 1). For costs equal or higher than 2 time steps, the work performed in a colony is reduced by 25%, compared to a population in the absence of switching costs.

**
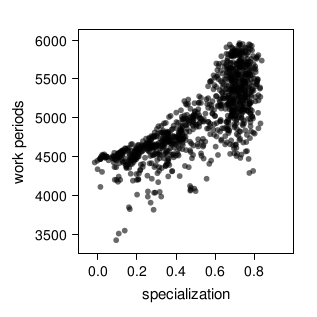
**

**Fig. S8** Relationship between the total amount of work performed in a colony and the degree of specialization within the colonies at generation 40,000 (*c* = 2).

As expected, mean specialization was highest for colonies showing highly differentiated thresholds for both tasks. In Fig. S9 we show the relationship between mean specialization obtained in colonies and the thresholds of colony parents, after 40000 generations. Colonies where parental thresholds (for one task or both) are similar achieve little or no specialization.

**
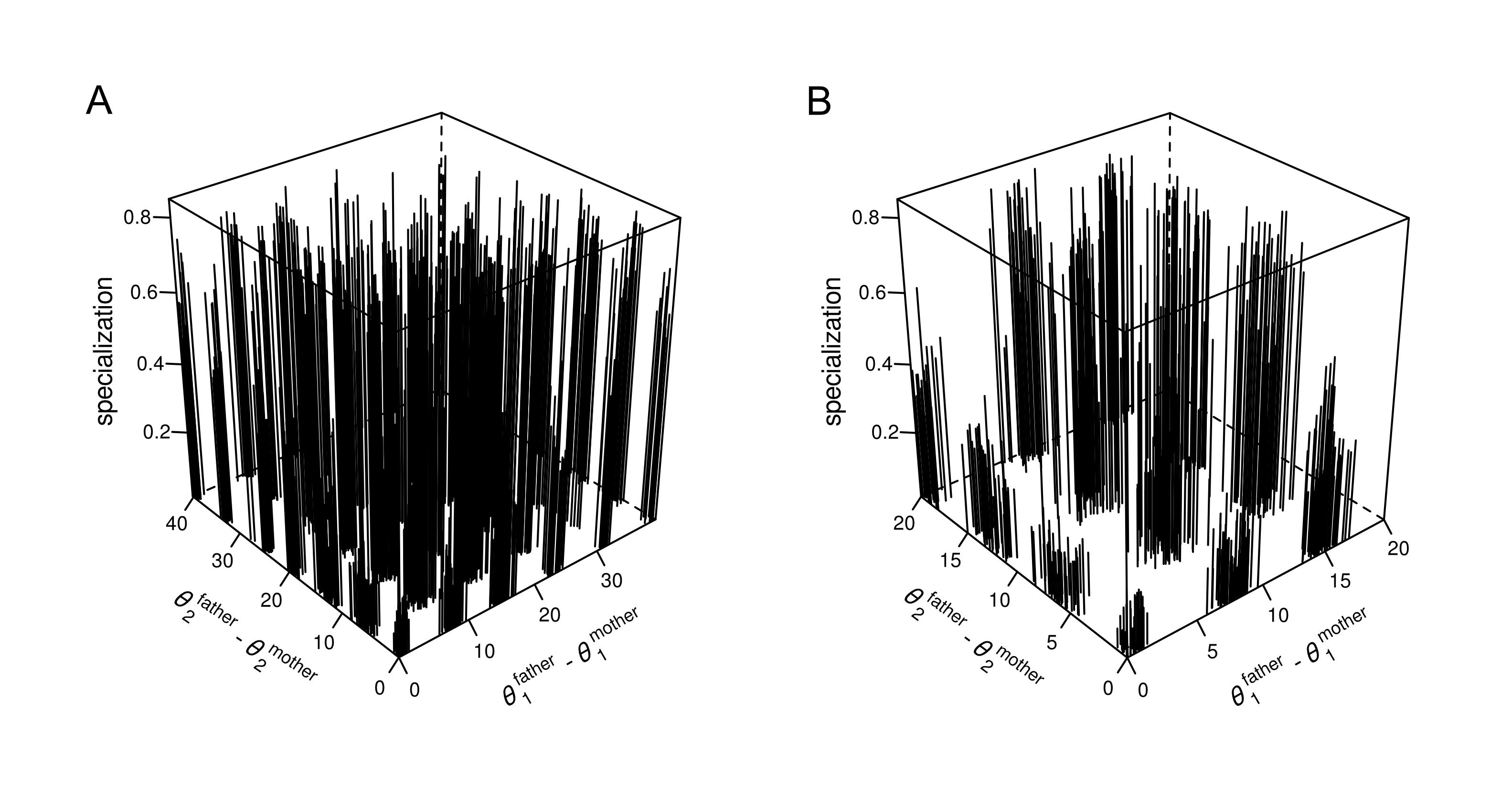
**

**Fig. S9** Relationship between colony specialization and the difference between parental thresholds for the two tasks. (A) Full data set of a typical simulation, after 40000 generations. (B) To make effects more visible, the subset of colonies is shown where the difference between parental thresholds ranges between 0 and 20. Colonies whose parents show larger differences in both their thresholds typically have higher specialization values.

At the end of each replicate simulation, we examined the behavior of the 10 colonies with highest resp. lowest degree of worker specialization, in order to understand what characterizes these colonies in terms of worker dynamics and threshold distribution. On average, colonies with a high degree of specialization have a larger number of active workers and reach the equilibrium number of workers of eq. (6) (Fig. S10a). Typically, colonies with high specialization have four types of workers, where the specialists have $\theta_{1}<<\theta_{2}$ or $\theta_{1}>>\theta_{2}$and the other two types have very similar thresholds, either high or low (illustrated in insets of Fig. Fig. S10Aa). Colonies without specialization do not show enough variation in thresholds to produce workers with different behavior (illustrated in inset of Fig. Fig. S10Bb). These colonies also stay below the number of workers needed for stimulus equilibrium, due to the fact that workers spend time being idle, while switching between tasks. Fig. S11 shows the frequency of worker performances for a typical colony with and without worker specialization. In the first case, a quarter of the individuals performs task 1 exclusively and at high frequency, while another quarter performs only task 2 exclusively and at high frequency (Fig. S11A). A part of the individuals (approximately 30%) does not perform either task. As for the colonies without worker specialization, very few individuals perform one of the tasks exclusively (Fig. S11B).

**
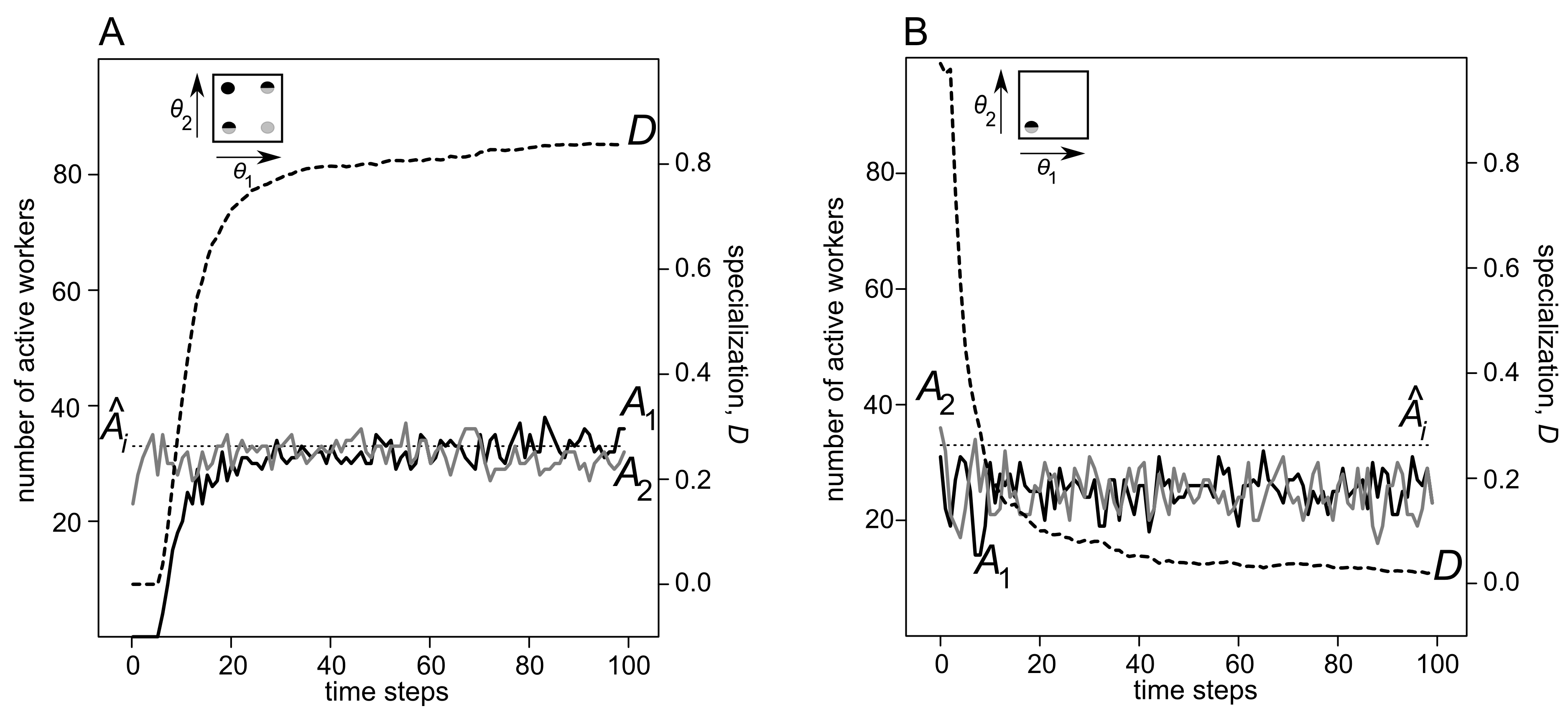
**

**Fig. S10** Worker dynamics of two extreme colonies from the last generation of the same simulation. (A) A colony with a high degree of specialization (hence, showing division of labor) and (B) a colony with a low degree of specialization (hence, no division of labor). On the left-hand *y*-axis, the number of active workers is shown, over simulation time steps (*x*-axis). The right-hand *y*-axis indicates the value of mean specialization in the colony (calculated as an average of the specialization values of all workers that are or were active). Insets in each graph illustrate the distribution of worker’s thresholds. Each circle represents a group of workers with similar thresholds. Colors within the circles indicate the tasks performed by the workers (black for task 1 and grey for task 2)


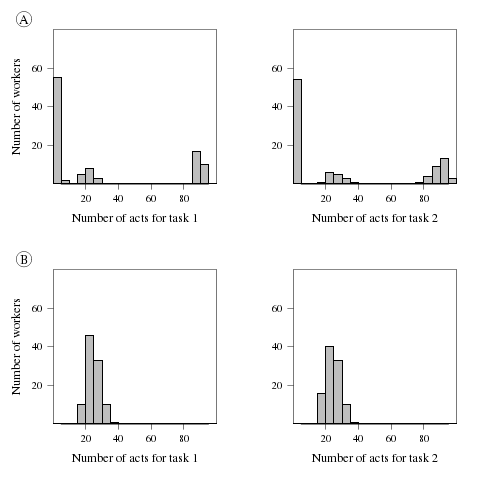


**Fig. S11** Histogram of worker performances for each task. Top graphs (A) correspond to the colony depicted in Fig. S10A, where a high degree of worker specialization is observed. Bottom graphs (B) correspond to the colony depicted in Fig. S10B, where worker specialization was very low

The results presented here and in the main text were highly repeatable across simulations. Fig. S12 shows, for different simulations, the distribution of mean specialization and worker distribution over colonies.


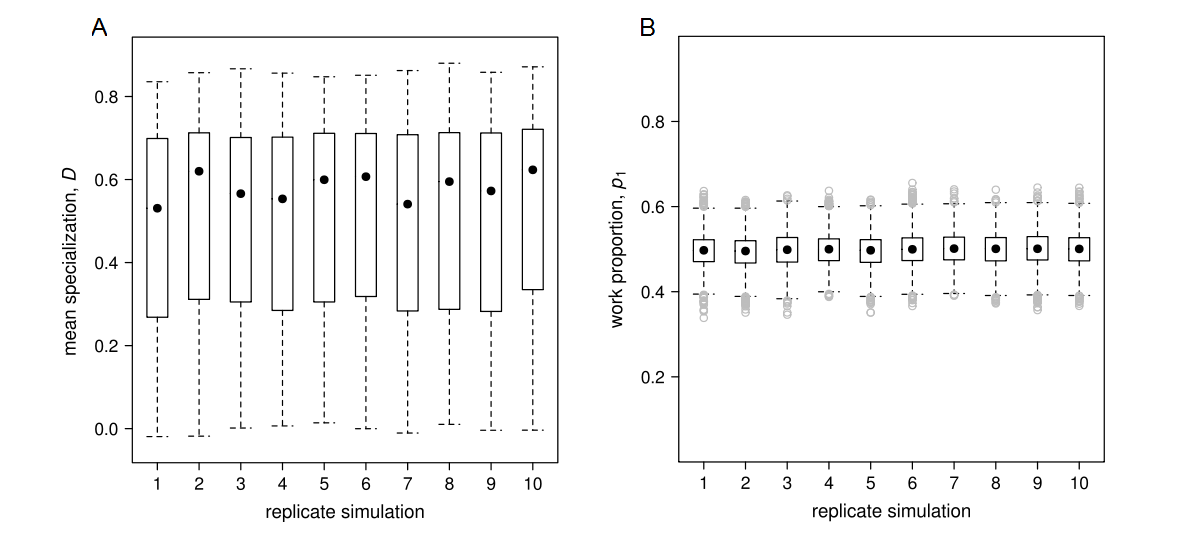


**Fig. S12** Distribution of (A) mean specialization, *D*, and (B) work proportion, $p_{1}$, across colonies that had evolved after 40,000 generations in different replicate simulations with *c* = 2. All parameter values are as in Fig. 2. Boxes show median (black closed circles) and interquartile range. Outliers are shown in grey open circles.

***Strong selection on worker distribution***

Under fitness scenario (4) it was not possible to evolve division of labor, when $\beta=0.5$. For low switching costs (1≤ *c* ≤ 2), the thresholds decreased to values near zero in all but one replicate, in which branching of the thresholds occurred. With increasing switching costs, threshold values did not drop to zero; in fact, for high switching costs (*c* ≥ 4), there was little change in thresholds throughout evolution (Fig. S13). It is likely that these results are due to the strong selection for an unbiased work distribution. As observed in the previous simulations, when specialization evolves the variance of *p*_1_ increases (for example, Fig. 4D in main text), due to the diversification of thresholds. Under fitness scenario (4) any deviation from the optimal work distribution is severely punished, hence populations cannot evolve specialization.


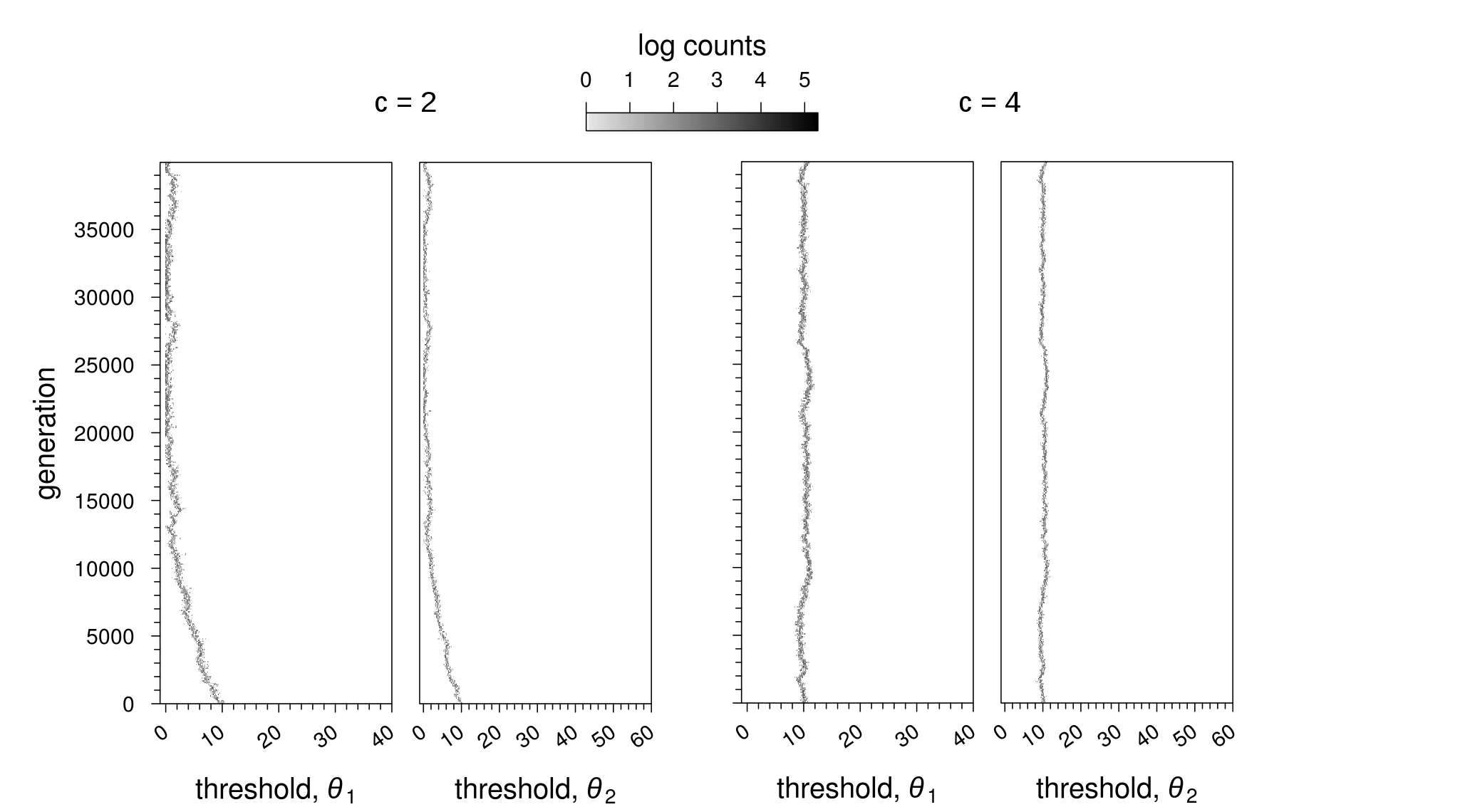


**Fig. S13** Evolutionary trajectories of thresholds in example simulations in which selection for worker distribution is strong (fitness scenario (4), $\beta=0.5$, $\sigma=0.1$). The presence of switching costs does not lead to branching of thresholds; with increasing switching costs, thresholds remain around their initial value of 10.

***Multiple mating***

The number of matings of female foundresses affected the evolutionary outcome under switching costs. When the number of matings, *m*, was low, specialization could evolve in all replicate simulations. However, the thresholds diversified into fewer branches (Fig. S14); comparing Fig. 4 in the main text and Fig. S14, which differ only in *m* (*m*=1 and *m*=2, respectively), we also observe that the level of specialization obtained was lower when m = 2, possibly because of lower differences between thresholds at the extreme of the distribution.


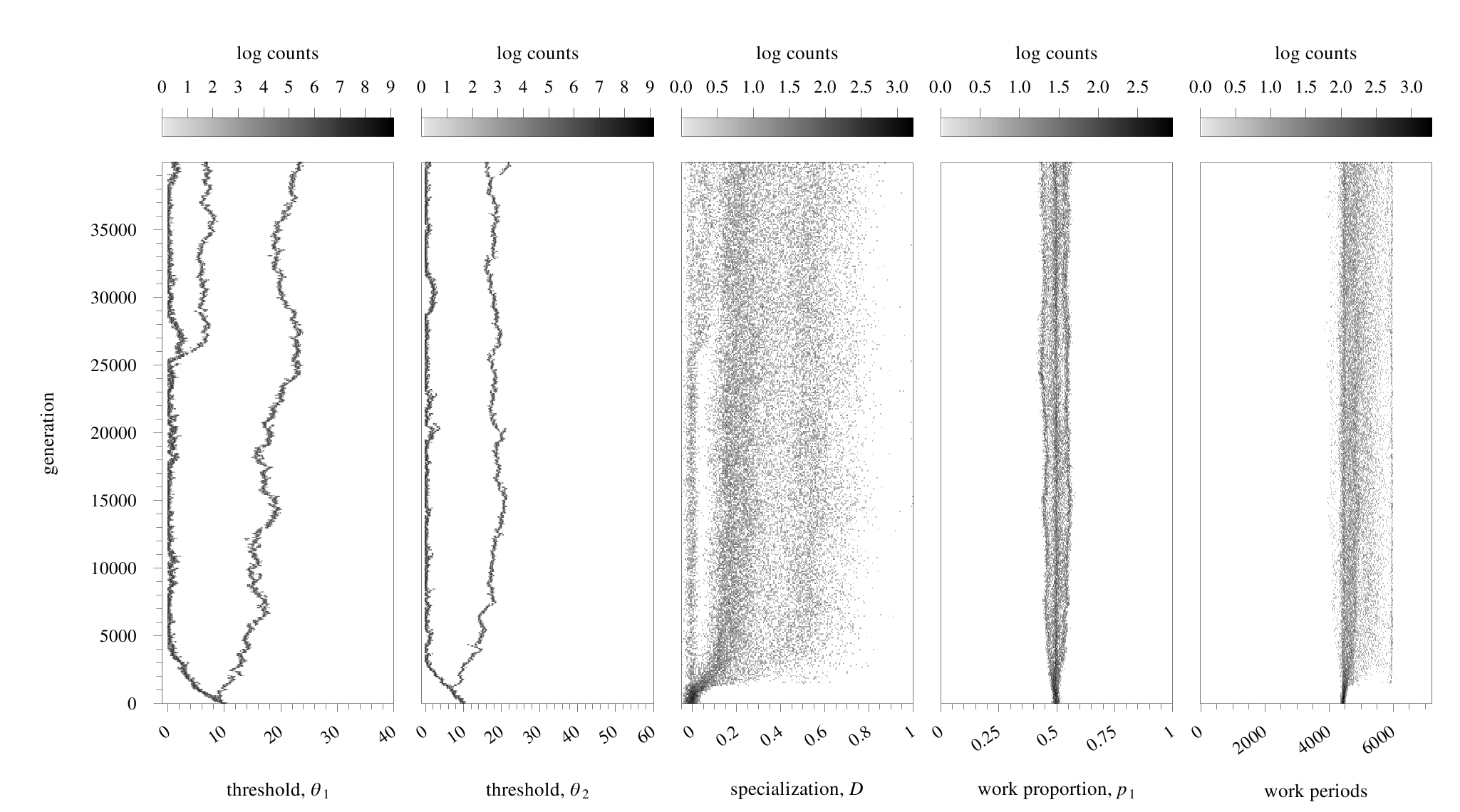


**Fig. S14** Evolutionary trajectories in example simulation with switching costs (*c* = 2), $\beta=0.5$and multiple mating (*m* = 2), under the standard fitness scenario (3).

**E. Effect of recombination**

In the main text, we assumed that the thresholds are inherited independently from each other ($r=0.5$). It is conceivable that division of labor and worker specialization gets off the ground more easily if the thresholds are linked, allowing the coadaptation of the two thresholds. To check for this, we also considered lower values of the recombination rate *r*. Here we only present some results for the case of complete linkage ($r=0$), for the model with single-mating and the standard fitness scenario (3).

In line with expectations, specialization can evolve already for relatively small switching costs ($c\geq1$, while $c\geq2$ was required in case of $r=0.5$). Fig. S15 shows a typical simulation with 1000 colonies. In contrast to the results in the main text, complete linkage allows maximal specialization ($D=1$) to be achieved by part of the population.

**
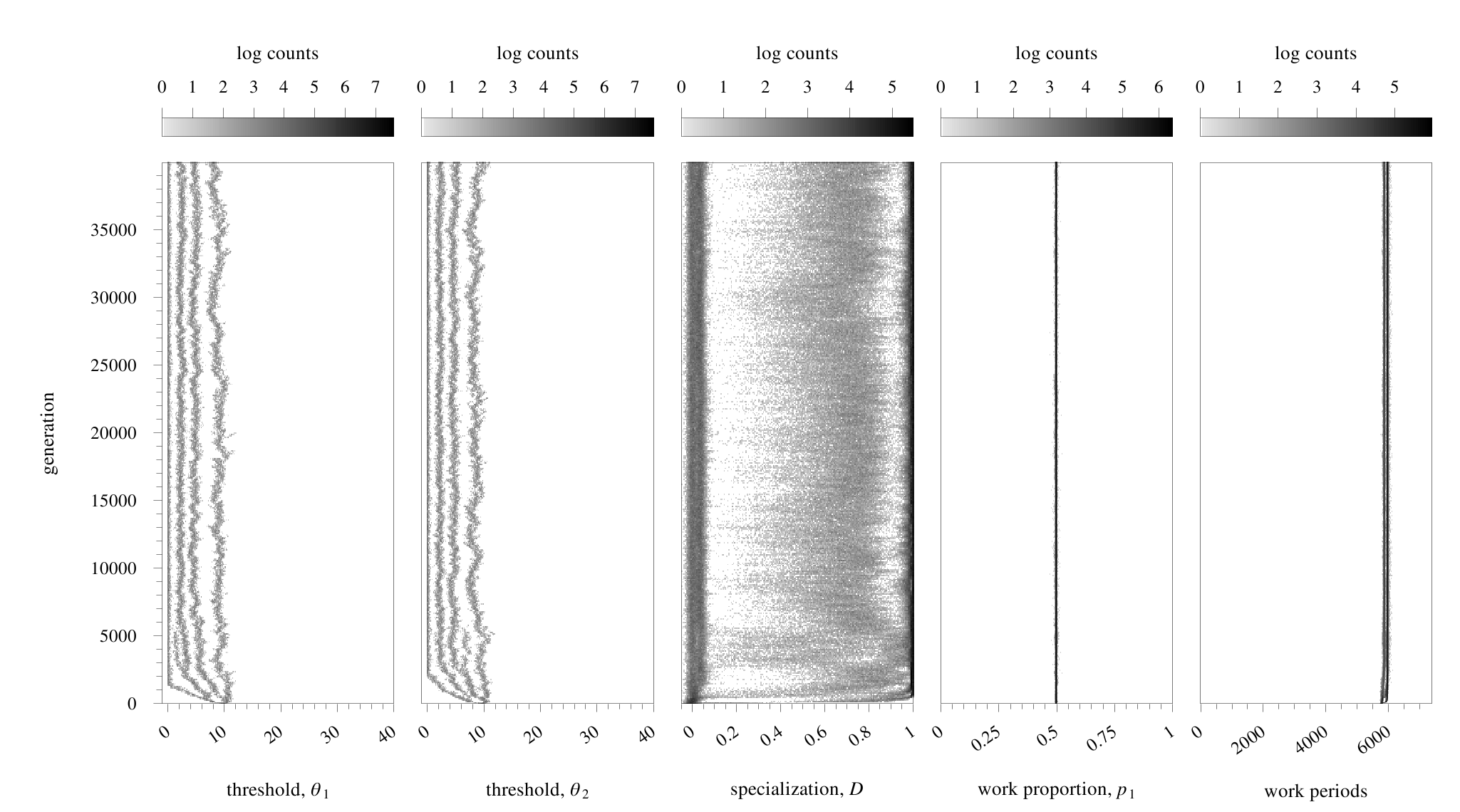
**

**Fig. S15** Evolutionary simulation of the response threshold model, for low switching costs ($c=1$) and complete linkage of the threshold loci ($r=0$).Graphical conventions, parameter values and fitness scenario as in Fig. 4 of the main text.

Linkage between the loci coding for the thresholds (*r* = 0) enables the thresholds for the two tasks to evolve as a single locus. This facilitates the evolution of specialization. The divergence of the thresholds was associated with the evolution of a strong negative correlation between thresholds (Fig. S16) – thus making it likely for individuals with a low threshold for one task to have a high threshold for the other task.

**
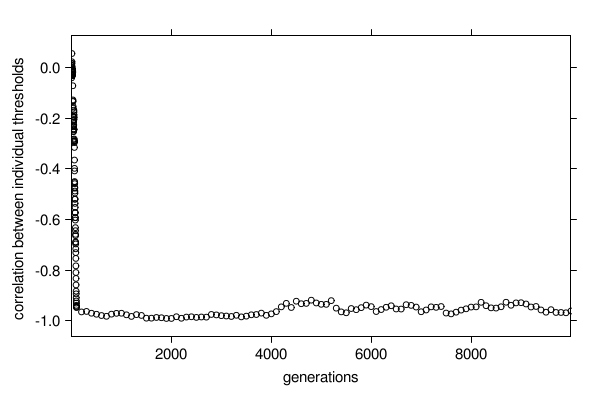
**

**Fig. S16** Correlation between individual thresholds in the simulation of Fig. S15. The correlation becomes strongly negative, indicating that a high threshold for one task is associated with a low threshold for the other.

**F. Effect of colony size**

We investigated whether colony size *N* has an effect on the evolutionary outcome of our model, by running simulations for different *N*. Here we show the results for *N* = 20, 50 and 500, under fitness scenario (3). The results show that colony size, in the current implementation of the response threshold model, does not affect the evolutionary outcome (Fig. S17). There is also no qualitative difference between these simulations and the ones ran for *N* = 100 (compare Fig. S17A and S17B with Figs. 2 and 4 in the main text, respectively). We have therefore chosen to only show in the main text the results for *N* = 100.


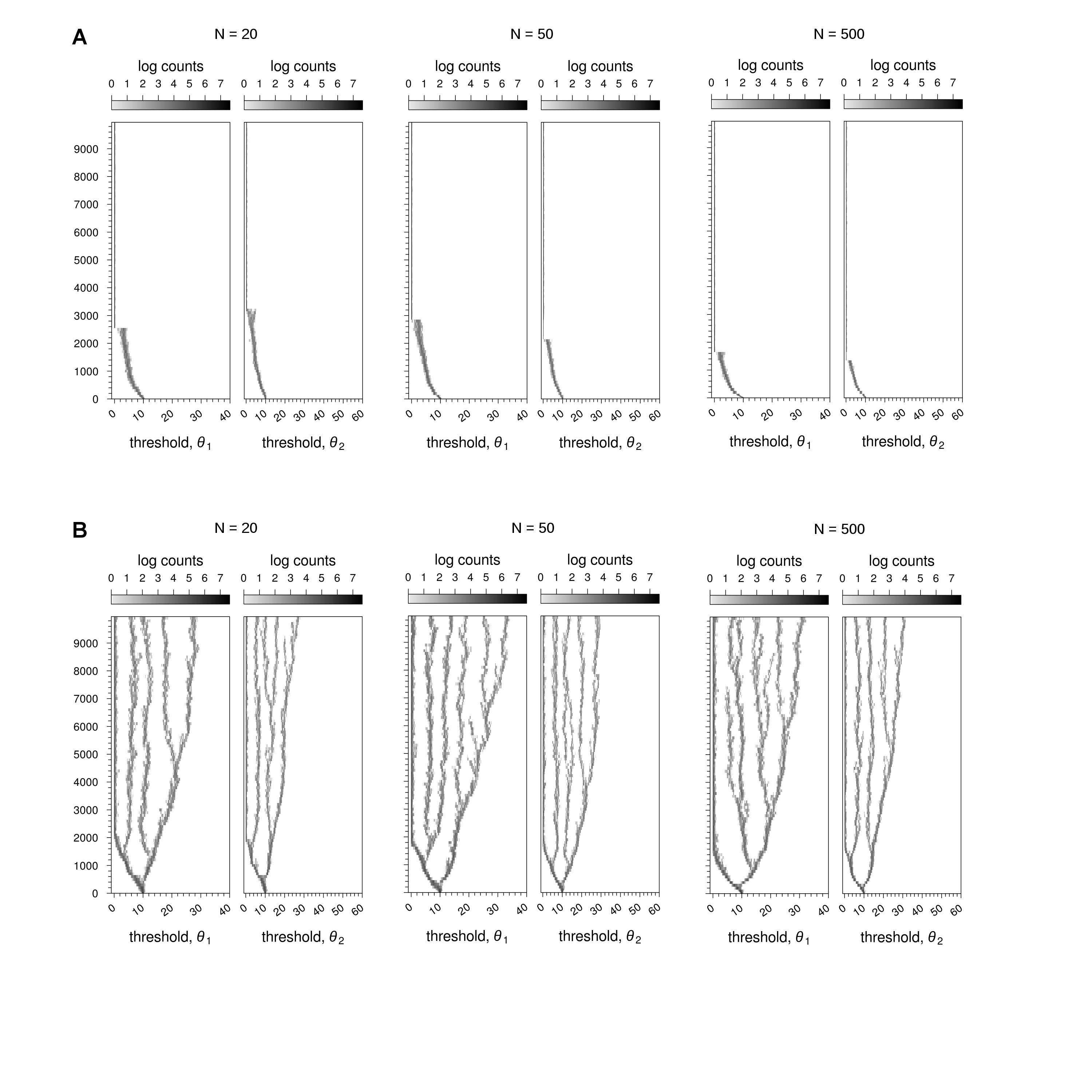


**Fig. S17** Evolutionary trajectories of thresholds for simulations with different colony size *N* (indicated above graphs). The simulations used the standard fitness scenario (3). (A) $\beta=0.75$and *c* = 0. (B) $\beta=0.5$and *c* = 2.

**References**

1. Bonabeau E, Theraulaz G, Deneubourg JL (1996) Quantitative study of the fixed threshold model for the regulation of division of labour in insect societies. Proc R Soc B*,* 263:1565-1569.

2. Abramowitz, M. and I. Stegun. 1972. Error Function and Fresnel Integrals *Handbook of Mathematical Functions with Formulas, Graphs, and Mathematical Tables*. Dover Publications, New York.
